# Driving in emergency medical service settings: a cross-sectional study of clinical psychological factors and perceived stress in a sample of ambulance drivers

**DOI:** 10.3389/fpubh.2026.1773290

**Published:** 2026-03-03

**Authors:** Pasquale Caponnetto, Paola Senia, Graziella Chiara Prezzavento, Eleonora Uccelli, Noemi Maria Vitale, Venerando Rapisarda, Francesco Alberto Fusco, Giuseppe Sferrazzo, Francesca Vella, Ermanno Vitale

**Affiliations:** 1 Section of Psychology, Department of Educational Sciences, University of Catania, Catania, Italy; 2 Department of Clinical and Experimental Medicine, University of Catania, Catania, Italy; 3 Department of Medicine and Surgery, University of Enna “Kore”, Enna, Italy

**Keywords:** ambulance drivers, burnout prevention, emergency medical services, occupational health, perceived stress, PSS-10

## Abstract

**Introduction:**

Ambulance drivers represent a critical yet understudied component of emergency healthcare systems, exposed to high levels of occupational stress. Despite their essential role in patient transport and emergency response, this population has received limited attention compared to other healthcare professionals. This cross-sectional study aimed to investigate perceived stress levels and associated factors among professional ambulance drivers, examining gender differences, predictive factors, and behavioral profiles.

**Methods:**

A sample of 1.436 ambulance drivers (76.7% male, mean age 52.77 years) underwent occupational health surveillance between October 2024–2025 at the Polyclinic University Hospital in Catania, Sicily. Perceived stress was assessed using the Perceived Stress Scale (PSS-10), which demonstrated excellent reliability (Cronbach’s *α* = 0.995). Data on sociodemographic characteristics, health status, and lifestyle habits were collected. Statistical analyses included independent *t*-tests, multiple linear regression, and cluster analysis.

**Results:**

Female drivers reported significantly higher perceived stress (*M* = 11.77, SD = 9.01) compared to males (*M* = 8.84, SD = 7.53), with a small-to-moderate effect size (Cohen’s *d* = −0.37). Multiple regression analysis identified several significant predictors of stress: female gender (*B* = 3.07, *p* < 0.001), longer work experience (*B* = 0.11, *p* = 0.006), presence of medical conditions (*B* = 1.22, *p* = 0.006), and higher alcohol consumption (*B* = 0.90, *p* = 0.017), while physical activity showed a protective effect (*B* = −0.28, *p* = 0.037). The model explained 4.5% of variance (*R*^2^ = 0.045). Cluster analysis revealed two distinct lifestyle profiles: one characterized by high physical activity but elevated alcohol consumption, and another by sedentary behavior with lower alcohol intake.

**Discussion:**

Ambulance drivers experience significant occupational stress influenced by multiple factors including gender, work seniority, health status, and lifestyle behaviors. Physical activity emerges as a protective factor, while alcohol consumption represents a risk factor. These findings highlight the need for multidimensional prevention strategies integrating both organizational interventions and individual support programs, with particular attention to gender-specific needs and promotion of healthy lifestyles.

## Introduction

Occupational stress represents a major public health concern, particularly among workers exposed to high cognitive, emotional, and organizational demands (1).

The work of ambulance drivers represents an essential component of territorial emergency systems and is characterized by high operational complexity and responsibility (1). This function requires not only specific technical skills, but also a continuous ability to manage critical situations, often associated with urgency, unpredictability, and significant responsibility in protecting patients’ lives. Numerous studies indicate that the health emergency context represents a high psychosocial risk work environment, in which workers, including drivers, may experience high levels of perceived stress, burnout, and impaired psychophysical wellbeing (2–4).

Ambulance drivers are exposed to multiple stressors: managing traumatic emergencies, intense emotional burdens, irregular schedules, and challenging environmental conditions. These factors, combined with patients’ responsibility for their lives, contribute to high psychological vulnerability (5–7).

Furthermore, recent evidence has shown that chronic stress and burnout in rescue workers are associated with sleep disturbances, worsening cognitive function, and increased risk of cardiovascular disease (8, 9).

Despite this evidence, the ambulance driver population remains relatively understudied compared to other healthcare professionals, such as doctors and nurses, despite similarly stressful working conditions (10). In particular, limited evidence is available on the combined role of perceived stress, individual characteristics, and work-related factors within an occupational health surveillance framework. In light of this, this study aims to analyze the levels of perceived stress in a large sample of ambulance drivers undergoing health surveillance. The specific objectives are: (a) to describe the distribution of stress in the population examined; (b) to analyze gender differences in PSS-10 scores; (c) to identify the main predictors of perceived stress through multiple regression; (d) to identify behavioral profiles through cluster analysis. This approach aims to outline a complex picture of the risk and protective factors associated with work-related stress, contributing to the international literature on the wellbeing of emergency workers (11).

## Materials and methods

### Study design

This study employed a cross-sectional observational design aimed at investigating perceived stress levels and associated sociodemographic, health, and lifestyle factors among ambulance drivers. Data were collected through structured assessments and standardized questionnaires during routine occupational health surveillance activities, in Italy mandatory by the law, in the period between October 2024 and October 2025.

It was not necessary to submit the study to the Ethics Committee for approval, since Italian law requires that such investigations be conducted to ensure the safety and health of workers.

### Participants and setting

The sample consisted of 1.436 ambulance drivers undergoing mandatory medical surveillance. All participants were employed in transportation roles and were assessed as part of their occupational health monitoring.

Inclusion criteria were: (1) employment as an ambulance driver, (2) participation in occupational health surveillance, and (3) completion of the Perceived Stress Scale (PSS). Exclusion criteria included incomplete data on key variables or refusal to consent to the processing of anonymous data for research purposes.

Data collection was carried out at the Occupational Medicine Unit of University of Catania (Sicily, Italy). The assessments were conducted during periodic medical examinations required by occupational safety law regulations.

### Measurements

Sociodemographic variables (age, gender, education), health status (presence of metabolic, endocrine, cardiovascular, or other conditions), and lifestyle habits (cigarette and alcohol consumption, physical activity, BMI) were recorded. Perceived stress was measured using the 10-item Perceived Stress Scale (PSS-10), which demonstrated excellent internal consistency (Cronbach’s alpha = 0.995). Work fitness status was also documented (e.g., suitable, suitable with limitations, temporarily or permanently unsuitable).

### Bias

To minimize selection bias, all eligible drivers undergoing routine evaluations during the study period were invited to participate. Information bias was reduced by using standardized instruments and trained personnel for data collection. However, self-reported lifestyle habits may be subject to reporting bias.

### Study size

The final sample included 1.436 participants, representing the total number of drivers assessed during the study period. No formal sample size calculation was performed, the study included the entire accessible population of ambulance drivers undergoing mandatory occupational health surveillance during the study period, thereby reducing selection bias. Participation rate exceeded 97%, further supporting the representativeness of the sample within this occupational setting.

### Statistical methods

All statistical analyses were conducted using SPSS version 29.0. Descriptive statistics were used to summarize sociodemographic, health, and lifestyle variables. Independent-samples *t*-tests were conducted to compare PSS scores between gender groups, with Levene’s test used to assess homogeneity of variances. Welch’s *t*-test was applied when variances were unequal. Effect sizes were calculated using Cohen’s d, Hedges’ g, and Glass’s delta.

Multiple linear regression was performed to identify predictors of perceived stress (PSS TOT), including age, gender, number of children, years of work, physical activity, cigarette and alcohol consumption, BMI, and health status. Model diagnostics included R^2^, Durbin–Watson statistic, and variance inflation factors (VIFs) to assess autocorrelation and multicollinearity.

Finally, a cluster analysis was conducted to explore lifestyle profiles based on standardized scores for cigarette and alcohol consumption, physical activity, and BMI. Two distinct clusters emerged, primarily differentiated by physical activity and alcohol use.

## Results

### Sociodemographic characteristics

Of a total of 1,474 workers who underwent the mandatory medical examination during the study period (1 year), 1,436 (97%) professional drivers participated in the study.

Of the 38 (100%) workers who did not participate in the study: 15 (39%) did not complete the questionnaire; 23 (61%) refused to consent to the processing of anonymous data for research purposes.

Most participants had completed secondary education and reported no relevant medical conditions. Among those reporting chronic diseases, cardiovascular conditions and metabolic disorders were the most frequently observed. Overall, the sample showed a predominantly sedentary lifestyle profile. Detailed sociodemographic, occupational, health, and lifestyle characteristics are reported in Table 1.

**Table 1:** Main characteristics of the sample.

Variables		Frequency (f)	Percentage (%)	Mean
Total sample		*N* = 1,436	100	–
Gender	F	334	23.3	–
	M	1,102	76.7	
Age	Total sample	1,436	100	52.77 ± 6.58
Seniority work	Total sample	1,436	100	15.14 ± 4.62
Educational level	University	94	6.55	–
	High school	841	58.56	
	Middle school	500	34.82	
	Elementary	1	0.07	
Work	Driver	1,436	100	–
Disease	Metabolic	87	6.06	–
	Endocrine	53	3.69	
	Heart	228	15.88	
	Mental	7	0.49	
	Other minor	98	6.82	
	No one	854	59.45	
Disease comorbidity	Two or more disorders	109	7.59	–
Lifestyle habits	Cigarette/day	–	–	3.73 ± 6.90
	Alcohol/day			0.22 ± 0.56
	Sport/week			1.61 ± 1.60
	BMI			27.38 ± 4.51
Suitable for work	Permanently unsuitable	6	0.42	–
	suitable	1,103	76.81	
	Suitable with prescription and limitation	314	21.86	
	Temporarily unsuitable	13	0.91	

### Reliability analysis

The Perceived Stress Scale (PSS-10) (12) demonstrated excellent internal consistency, with Cronbach’s alpha = 0.995 across the 10 items, confirming the reliability of the instrument in this population.

### Gender differences in perceived stress

An independent-samples *t*-test was conducted to compare perceived stress scores (PSSTOT) between genders (see Table 2). Perceived stress scores differed significantly between male and female ambulance drivers. Due to the violation of homogeneity of variances, group comparisons were performed using Welch’s *t*-test, which revealed a statistically significant difference in perceived stress levels between genders (*p* < 0.001). Female drivers reported higher perceived stress compared to their male counterparts.

**Table 2:** *T*-test independent sample.

Variable	Variance assumption	Levene’s test for equality of variances		*t*-test for equality of means						
		*F*	Sig.	*T*	df			Mean difference		
						Sig. (1-tailed)	Sig. (2-tailed)		95% CI lower	95% CI upper
PSSTOT	Equal variances assumed	19.113	<0.001	−5.95	1,434	<0.001	<0.001	−2.94 ± 0.049	−3.90	−1.969
	Equal variances not assumed			−5.41	482.36	<0.001	<0.001	−2.94 ± 0.54	−4.00	−1.87

Although statistically robust, the magnitude of this difference was small-to-moderate, indicating that gender alone accounted for a limited proportion of the overall variability in perceived stress. This finding suggests that, while gender represents a relevant factor in stress perception among ambulance drivers, additional individual, occupational, and lifestyle-related variables likely play a substantial role in shaping stress levels. Detailed statistical results and effect size estimates are reported in Tables 2, 3.

**Table 3:** Effect size for independent sample.

Variable	Effect size	Standardizer^a^	Point estimate	95% CI lower	95% CI upper
PSSTOT	Cohen’s d	7.894	−0.37	−0.49	−0.25
	Hedges’ g	7.898	−0.37	−0.49	−0.25
	Glass’s delta	9.005	−0.33	−0.45	−0.21

The denominator used to estimate effect sizes differs: Cohen’s d uses the pooled standard deviation; Hedges’ g applies a correction factor; Glass’s delta uses the standard deviation of the control group. ^a^The denominator used to estimate the effect size.

### Multiple linear regression

Multiple linear regression analysis was performed to identify independent predictors of perceived stress among ambulance drivers. The overall model was statistically significant (*p* < 0.001), although it explained a relatively small proportion of variance, indicating a multifactorial stress profile not fully captured by the variables included in the model.

Female gender, longer work seniority, presence of medical conditions, and higher alcohol consumption were independently associated with higher perceived stress levels, whereas greater engagement in physical activity showed a protective association. In contrast, age, number of children, cigarette consumption, and body mass index were not significantly related to perceived stress.

Model diagnostics indicated adequate statistical robustness, with no evidence of multicollinearity and no residual autocorrelation.

Other variables (age, number of children, cigarette consumption, BMI) were not significant predictors. No multicollinearity issues were detected (all VIF < 1.11), and residuals showed no evidence of autocorrelation (Durbin–Watson = 1.82). The model identified several significant predictors, although the explained variance was modest (*R*^2^ = 0.045), indicating that other unmeasured factors likely contribute to perceived stress (see Table 4).

**Table 4:** Multiple linear regression predicting perceived stress (PSSTOT).

Predictor	Non-standardized coefficients	Standardized coefficients	*t*	*P*	95% CI lower	95% CI upper	Collinearity statistics	
	B	Beta					Tolerance	VIF
Constant	8.34 ± 2.24	–	3.730	<0.001	3.956	12.733		
Age	−0.05 ± 0.03	−0.040	−1.421	0.155	−0.115	0.018	0.856	1.168
Gender	3.07 ± 0.51	0.163	6.080	<0.001	2.082	4.065	0.936	1.068
Children numbers	−0.24 ± 0.17	−0.037	−1.422	0.155	−0.579	0.092	0.983	1.017
Age of work	0.11 ± 0.04	0.074	2.750	0.006	0.031	0.188	0.914	1.094
Sport/week	−0.28 ± 0.13	−0.056	−2.088	0.037	−0.544	−0.017	0.928	1.077
Cigarettes/day	0.03 ± 0.03	0.028	1.057	0.291	−0.028	0.092	0.971	1.030
Alcohol/day	0.90 ± 0.37	0.063	2.394	0.017	0.162	1.635	0.956	1.047
BMI	0.02 ± 0.05	0.013	0.489	0.625	−0.070	0.116	0.936	1.069
Health status*	1.22 ± 0.44	0.075	2.745	0.006	0.348	2.091	0.903	1.107

Model summary: *R* = 0.213; *R*^2^ = 0.045; Adjusted *R*^2^ = 0.039; *F* (9, 1,426) = 7.50, *p* < 0.001; Durbin–Watson = 1.824. *Health status was dummy-coded (0 = no relevant medical conditions; 1 = presence of one or more medical conditions). Unstandardized coefficients (B) represent the expected change in PSSTOT associated with a one-unit increase in the predictor, holding all other variables constant.

### Cluster analysis of lifestyle profiles

A two-cluster solution was identified based on lifestyle indicators (alcohol consumption, cigarette use, physical activity, and BMI) (see Table 5). The first cluster was characterized by markedly higher levels of physical activity but also substantially higher alcohol consumption. In contrast, the second cluster included participants with lower levels of physical activity and lower alcohol intake. No meaningful differences emerged between clusters in terms of smoking behavior or BMI. Thus, the primary axis of differentiation within the sample was defined by physical activity and alcohol consumption, rather than by smoking or body weight. The main lifestyle distinction was between a physically active but higher alcohol-consuming group and a more sedentary group with lower alcohol intake, while smoking and BMI did not differentiate clusters.

**Table 5:** Cluster analysis of lifestyle variables.

Variables (Z-scores)	Cluster		F (descriptive)	P (descriptive)
	1	2		
Alcohol/day	17.36426	−0.01210	381.80	<0.001
Cigarettes/day	−0.39547	0.00028	0.16	0.693
Sport/week	5.25148	−0.00366	28.12	<0.001
BMI	0.37271	−0.00026	0.14	0.709

*F*-tests are descriptive only. as clusters were defined to maximize between-group differences. Significance levels cannot be interpreted as hypothesis tests.

## Discussion

The results of this study confirm that ambulance drivers represent a category at high risk of stress and burnout. Perceived stress levels vary significantly between subjects, with women reporting higher scores, a finding consistent with what has been observed in other studies of emergency workers (4, 13). Recent evidence from international contexts corroborates these gender-based differences. A cross-sectional study conducted in India during the COVID-19 pandemic found that female ambulance drivers and other healthcare workers experienced significantly higher levels of psychological distress compared to males, with distress associated with longer work hours, changes in work schedules, and direct involvement in pandemic response activities (14, 15). This study specifically identified longer hours of work (≥8 h/day), direct COVID-19 patient care, and high emotional exhaustion as significant risk factors for psychological distress among healthcare workers, including ambulance drivers (16).

Research examining female emergency medical services clinicians more broadly has documented that women face heightened risk for adverse mental health outcomes, including anxiety, depression, and post-traumatic stress disorder.

A recent cross-sectional study of Emergency Medical Service (EMS) clinicians reported rates of 34% for anxiety and 29.2% for depression among EMS personnel (17). Another mixed-methods study found that female EMS clinicians had high rates of probable anxiety (70.0%), probable depression (53.9%), and elevated burnout (40.9%), with workplace harassment and lack of support being common experiences (18) These disparities are influenced not only by the inherent stressors of emergency work but also by gender-based challenges in the workplace, including discrimination, lack of support, and concerns about appearing incompetent. Qualitative and quantitative data from female EMS clinicians highlight that discrimination, harassment, and insufficient workplace support contribute to adverse mental health outcomes and increased occupational burden (19). Additionally, lower leadership support and greater work-related concerns have been shown to mediate the increased risk of chronic stress-related psychological sequelae in women.

Among the main predictors of perceived stress identified in our study are seniority, health problems, alcohol consumption, and poor physical activity, all factors already highlighted in the international literature as determinants of psychological wellbeing (5, 20, 21). The relationship between work seniority and stress is particularly noteworthy. While experience typically serves as a protective factor, prolonged exposure to high-stress emergency situations can lead to cumulative trauma and emotional exhaustion. Recent findings from a Chinese study of 213 ambulance drivers demonstrated that occupational stress was positively correlated with burnout and negatively associated with sleep quality, with burnout partially mediating the relationship between occupational stress and sleep disturbances. This mediation effect accounted for 26.09% of the total effect, suggesting that chronic stress operates through burnout to impair essential recovery processes (2). Frontline ambulance staff in the United Kingdom, particularly paramedics and emergency medical technicians (EMTs), are most affected by burnout, with high prevalence and severity of both burnout and depersonalization. Recent systematic reviews and qualitative studies indicate that more than half of UK ambulance personnel experience significant levels of burnout, and up to 87% report moderate or high levels of depersonalization toward their work (22). These findings are consistent with broader evidence that occupational stress and burnout are highly prevalent among ambulance personnel, and that sleep quality is both a consequence and mediator of these relationships.

Perceived stress therefore proves to be a multifactorial phenomenon, influenced by individual, behavioral, and organizational elements. Physical activity emerges as a protective factor in our study, while alcohol consumption aggravates stress, findings that align with results obtained from studies on prehospital staff (11, 23). The protective role of physical activity may operate through multiple mechanisms, including stress hormone regulation, improved sleep quality, and enhanced psychological resilience (24, 25). Alcohol consumption as a coping mechanism may worsen stress responses and mental health. Longitudinal cohort data from UK healthcare workers show that increased alcohol use is associated with higher symptoms of depression, anxiety, and PTSD, particularly when used as a coping strategy in response to occupational stressors (26). Additional evidence from ambulance personnel indicates that drinking to cope is a significant risk factor for alcohol-related problems and is associated with higher levels of depersonalization and burnout (27). Neurobiological studies further support that stress and alcohol have bidirectional effects on brain networks involved in emotional processing, contributing to comorbid psychiatric illness and alcohol use disorder (28). A review examining the effect of ambulance clinicians’ wellbeing on occupational and patient safety identified that burnout, stress, poor sleep quality, and fatigue increase the risk of accidents and adverse events. The review underscored that these factors not only compromise provider wellbeing but also pose direct threats to patient safety and occupational safety in prehospital emergency medical services settings (29).

Organizational conditions, such as workload and supervisor support, significantly influence burnout risk and resilience (3, 10). The statement that 73% of EMS providers nationwide report burnout or compassion fatigue is supported by recent survey-based studies and systematic reviews, which consistently demonstrate high prevalence of burnout and compassion fatigue among U. S. EMS personnel, with rates ranging from 39% to over 70% depending on the measurement tool and population sampled (30, 31). The finding that 37% plan to leave the field within 5 years is corroborated by national cross-sectional analyses showing that intention to leave EMS within 1–5 years is common, particularly among those working overtime or multiple jobs, with financial dependence and job dissatisfaction as key drivers (32, 33). Nearly 60% of EMS agencies report insufficient staffing to meet emergency call demands is reflected in workforce studies indicating widespread staffing shortages and turnover, with stress and burnout as major contributors to attrition and inability to meet operational needs (32). 49% of paramedics work more than 40 h per week and many holds multiple jobs is substantiated by large-scale survey data showing that the majority of EMS professionals work over 40 h weekly, and a substantial proportion rely on overtime or multiple jobs to make ends meet (33). Only 27% of EMS providers meet recommended sleep guidelines, and 61% cite lack of time as a barrier to maintaining physical health is supported by systematic reviews and occupational health studies documenting poor sleep quality, high rates of fatigue, and lack of time for health maintenance among EMS clinicians (29). Recent studies on EMS workforce retention have identified burnout and stress as major contributors to high sickness rates and staff attrition, with the first 2 years of employment being particularly critical for retention. Stress and burnout are the most frequently cited reasons for leaving EMS, and younger, less experienced staff are at highest risk for early attrition (34). Evidence from intervention studies indicates that coping training programs can reduce emotional exhaustion levels among ambulance staff, with more modest effects on depersonalization and personal accomplishment. A systematic review of interventions targeting resistance and resilience among EMS clinicians found that mindfulness-based and coping-focused interventions were associated with reductions in burnout, particularly emotional exhaustion, at up to 6 months of follow-up, though effects on depersonalization and personal accomplishment were less pronounced (35). A quasi-experimental study of the FIRECARE program, which combined mindfulness, heart coherence, and positive psychology training for advanced life support ambulance staff, demonstrated significant reductions in burnout, primarily in emotional exhaustion, at 3 months post-intervention, with smaller or non-significant changes in depersonalization and personal accomplishment (36). These findings are consistent with broader evidence that resilience and coping interventions for healthcare professionals, including ambulance staff, are most effective in reducing emotional exhaustion, with more limited impact on other burnout dimensions. Gender-specific considerations warrant particular attention in prevention and intervention strategies. Women in EMS face unique challenges that extend beyond the inherent stressors of emergency work. Studies examining female EMS experiences have documented higher rates of workplace discrimination, sexual harassment, and concerns about being perceived as incompetent (18). Female paramedics report being 28.4% more likely than their male counterparts to fear appearing incompetent and therefore less likely to speak openly about mistakes, which can have significant implications for both learning and patient safety (37). Research from Turkey, where women comprise 72% of paramedics, reveals that female providers face gender discrimination, violence, and professional challenges that adversely affect their personal and professional wellbeing (18, 38). The intersection of gender-based stressors with the already demanding nature of emergency work creates a compounded risk for psychological distress among female ambulance personnel. Addressing these gender-specific challenges requires not only individual support but also systemic changes to workplace culture and leadership practices.

The mental health crisis within emergency medical services is further evidenced by data on post-traumatic stress disorder prevalence. A study from Saudi Arabia found that 26% of EMS personnel screened positive for PTSD, with exposure to traumatic events being a significant contributing factor. Interestingly, research suggests that PTSD in paramedics is not always caused by major traumatic incidents but can arise from smaller everyday events that trigger symptoms, combined with chronic organizational stressors such as excessive workload, pressure from management, and changing shift patterns (39). This finding emphasizes that prevention efforts must address both acute traumatic exposures and chronic organizational stressors. The prevalence of mental health issues among EMS personnel has profound implications not only for individual wellbeing but also for patient care quality, as psychological distress is associated with increased risk of clinical errors, communication failures, diminished clinical capability, absenteeism, and increased turnover.

Recent studies confirm that resilience, functional coping, and ongoing psychological support are key tools for preventing burnout and improving service quality (40, 41). However, access to mental health support remains inadequate. Barriers to accessing support include stigma, lack of time, and insufficient availability of services tailored to the unique needs of emergency personnel (42). Expanding mental health services and reducing barriers to access represent critical priorities for EMS organizations. Additionally, research has shown that supportive workplace policies, such as adequate paid sick leave, are associated with better job satisfaction and retention, suggesting that comprehensive benefits packages may serve as both a recruitment and retention strategy while supporting employee wellbeing. The findings of this study may have important implications for public health and occupational prevention. The observed associations are consistent and may translate into a significant population-level impact, considering large working populations and long latency periods. These findings support the role of primary prevention strategies aimed at reducing occupational exposures through exposure-oriented risk assessment, source control, and organizational interventions. These findings can contribute to risk prioritization and guide occupational preventive actions. Furthermore, future research should focus on longitudinal study designs with improved exposure assessment, including cumulative and mixed exposures, as well as the integration of biological markers and individual susceptibility factors to better elucidate causal pathways and improve risk stratification.

This study presents several limitations that warrant consideration. The reliance on self-reported measures introduces potential recall and social desirability biases, particularly concerning alcohol consumption, smoking habits, and physical activity. The PSS-10, while psychometrically sound, captures only subjective stress appraisal without objective physiological indicators. The single-center setting in Sicily limits generalizability to ambulance drivers in other geographic regions or healthcare systems. The sample consisted exclusively of drivers undergoing mandatory health surveillance, potentially introducing survivor bias by excluding those who had already left the profession due to stress. The modest explained variance in the regression model (*R*^2^ = 0.045) indicates that unmeasured factors such as organizational climate, social support, specific traumatic exposures, and coping resources likely contribute substantially to stress levels.

Despite these limitations, this study provides valuable preliminary insights into stress profiles and identifies potentially modifiable risk factors for occupational health interventions: first, the high prevalence of stress and burnout among ambulance drivers necessitates routine psychological screening and early intervention programs. Proactive identification of at-risk individuals, combined with timely access to support services, may prevent the progression from stress to clinical burnout and mental health disorders. Second, organizational interventions must address modifiable workplace factors, including workload management, shift scheduling, adequate staffing levels, and supportive leadership practices. Recent evidence suggests that organizational commitment, peer support, and authentic leadership can attenuate work-related stressors among EMS clinicians. Third, gender-sensitive approaches to workplace policy and culture are essential, given the documented disparities in stress, mental health outcomes, and workplace experiences between male and female EMS personnel. The observed also that gender distribution, with a predominance of male participants, reflects the current occupational composition of ambulance drivers in the Italian Emergency Medical Services, where driving roles are still predominantly held by men. Future studies conducted in different organizational and cultural settings are needed to confirm these results. Leadership development programs that emphasize transformational and inclusive leadership styles may be particularly valuable in creating more equitable and supportive work environments.

## Conclusion

The evidence collected highlights the need to implement multidimensional prevention strategies that integrate organizational (shifts, support, work climate) and individual (physical activity, reduction of alcohol consumption, coping training) interventions, with particular attention to gender differences and health factors. Given the documented workforce crisis, with substantial proportions of EMS personnel considering leaving the profession, urgent action is required at multiple levels, from local service provision to national policy. Future research should employ longitudinal designs to better understand the temporal relationships between occupational stressors, mental health outcomes, and workforce retention, as well as to evaluate the effectiveness of prevention and intervention programs in real-world EMS settings. Only through comprehensive, evidence-based approaches that address both individual and systemic factors can we hope to sustain a healthy, resilient emergency medical services workforce capable of providing high-quality care to communities.

## Data Availability

The raw data supporting the conclusions of this article will be made available by the authors, without undue reservation.
